# Anthropometric Indicators and Early Cardiovascular Prevention in Children and Adolescents: The Role of Education and Lifestyle

**DOI:** 10.3390/jcdd13010057

**Published:** 2026-01-22

**Authors:** Elisa Lodi, Maria Luisa Poli, Emanuela Paoloni, Giovanni Lodi, Gustavo Savino, Francesca Tampieri, Maria Grazia Modena

**Affiliations:** 1 CHIMOMO Department, University of Modena e Reggio Emilia, Via del Pozzo, 71, 41124 Modena, Italy; 2 Centro P.A.S.C.I.A. (Programma Assistenziale Scompenso Cardiaco, Cardiopatie dell’Infanzia e A Rischio) AOU Policlinico di Modena, Via del Pozzo 71, 41121 Modena, Italy; 3 Servizio di Medicina dello Sport, Azienda USL di Modena, Via Rita Levi Montalcini, 60, 41122 Modena, Italy

**Keywords:** pediatric obesity, waist-to-height ratio, cardiovascular prevention, public health prevention, pediatric cardiometabolic risk, health education

## Abstract

Background: Childhood obesity represents the most common nutritional and metabolic disorder in industrialized countries and constitutes a major public health concern. In Italy, 20–25% of school-aged children are overweight and 10–14% are obese, with marked regional variability. Excess adiposity in childhood is frequently associated with hypertension, dyslipidemia, insulin resistance, and non-alcoholic fatty liver disease (NAFLD), predisposing to future cardiovascular disease (CVD). Objective: To investigate anthropometric indicators of cardiometabolic risk in 810 children and adolescents aged 7–17 years who underwent assessment for competitive sports eligibility at the Sports Medicine Unit of Modena, evaluate baseline knowledge of cardiovascular health aligned with ESC, AAP (2023), and EASO guidelines. Methods: 810 children and adolescents aged 7–17 years undergoing competitive sports eligibility assessment at the Sports Medicine Unit of Modena underwent evaluation of BMI percentile, waist circumference (WC), waist-to-height ratio (WHtR), and blood pressure. Cardiovascular knowledge and lifestyle habits were assessed via a previously used questionnaire. Anthropometric parameters, blood pressure (BP), and lifestyle-related knowledge and behaviors were assessed using standardized procedures. Overweight and obesity were defined according to WHO BMI-for-age percentiles. Elevated BP was classified based on the 2017 American Academy of Pediatrics age-, sex-, and height-specific percentiles. Statistical analyses included descriptive statistics, group comparisons, chi-square tests with effect size estimation, correlation analyses, and multivariable logistic regression models. Results: Overall, 22% of participants were overweight and 14% obese. WHtR > 0.5 was observed in 28% of the sample and was more frequent among overweight/obese children (*p* < 0.001). Elevated BP was detected in 12% of participants with available measurements (*n* = 769) and was significantly associated with excess adiposity (χ^2^ = 7.21, *p* < 0.01; Cramér’s V = 0.27). In multivariable logistic regression analyses adjusted for age and sex, WHtR > 0.5 (OR 2.14, 95% CI 1.32–3.47, *p* = 0.002) and higher sedentary time (OR 1.41 per additional daily hour, 95% CI 1.10–1.82, *p* = 0.006) were independently associated with elevated BP, whereas BMI percentile lost significance when WHtR was included in the model. Lifestyle knowledge scores were significantly lower among overweight and obese participants compared with normal-weight peers (*p* < 0.01). Conclusions: WHtR is a sensitive early marker of cardiometabolic risk, often identifying at-risk children missed by BMI alone. Baseline cardiovascular knowledge was suboptimal. The observed gaps in cardiovascular knowledge underscore the importance of integrating anthropometric screening with structured educational interventions to promote healthy lifestyles and long-term cardiovascular prevention.

## 1. Introduction

Childhood obesity has escalated to epidemic levels worldwide and is increasingly acknowledged as a significant factor influencing long-term cardiovascular health issues [1,2,3,4]. The excess weight gained during childhood tends to persist into adulthood, leading to the premature onset of hypertension, dyslipidemia, insulin resistance, and changes in cardiovascular structure. Notably, atherosclerotic changes can begin early in life, often before any clinical signs of cardiovascular disease appear, underscoring the vital need for early detection and preventive measures. In Europe, and especially in Italy, the rates of pediatric overweight and obesity remain alarmingly elevated despite years of public health efforts [1,2,3]. National surveillance statistics reveal that around one in three children in Italy is affected by excess body weight, with marked regional and socioeconomic differences. These patterns raise alarms not only regarding the future burden of cardiovascular diseases but also concerning the early onset of cardiometabolic issues during childhood and adolescence.

Pediatric obesity is a diverse condition rather than a uniform one [5,6]. Central adiposity is crucial in the development of early cardiometabolic risks. Visceral fat tissue is metabolically active and fosters chronic low-grade inflammation by increasing the secretion of pro-inflammatory cytokines and modifying adipokine profiles, which leads to insulin resistance, endothelial dysfunction, arterial stiffness, and ectopic fat accumulation [7,8]. These processes hasten vascular aging and can result in early increases in blood pressure and cardiac remodeling, even among young individuals [9,10]. Anthropometric measurements serve as straightforward, cost-effective, and broadly applicable methods for early risk assessment. While BMI percentile is the most frequently utilized metric in pediatric settings, it fails to adequately reflect fat distribution or visceral fat levels. Conversely, the waist-to-height ratio (WHtR) offers a dependable and age-independent assessment of central fat accumulation and has demonstrated stronger correlations with cardiometabolic risk factors, such as hypertension, dyslipidemia, insulin resistance, and subclinical atherosclerosis [10,11,12,13,14,15].

Nevertheless, during puberty, physiological changes in body composition and fat distribution—particularly the transient increase in central adiposity observed in early and mid-adolescence—may influence anthropometric measurements and risk thresholds, underscoring the importance of interpreting these indices within a developmental context [16]. A WHtR threshold of 0.5 has been suggested as a practical limit for identifying heightened cardiometabolic risk in children and adolescents. In addition to biological risk factors, lifestyle behaviors and cardiovascular health literacy significantly influence early risk trajectories. Physical inactivity, excessive sedentary behavior, and poor dietary habits often co-occur with obesity and central adiposity. Educational interventions that emphasize nutrition and physical activity have shown positive effects on both knowledge and cardiometabolic profiles [15,17,18,19]. Nevertheless, there is a scarcity of data that combines anthropometric screening with cardiovascular education in Italian pediatric populations. Children who are being assessed for competitive sports eligibility represent a distinct and under-researched group. While they are frequently viewed as healthy and physically active, cardiometabolic risk factors may go unnoticed without specific screening. Sports medicine evaluations provide a structured and recurring opportunity for interaction with healthcare professionals, thus serving as an underutilized chance for early risk identification and cardiovascular education. The current study aimed to evaluate anthropometric indicators of cardiometabolic risk and baseline cardiovascular health knowledge in a large group of children and adolescents undergoing competitive sports eligibility assessment. By merging biological and behavioral aspects, this study intends to emphasize the significance of WHtR as an early indicator of cardiometabolic vulnerability and to highlight the necessity of education in pediatric cardiovascular prevention.

## 2. Methods

A cross-sectional study was carried out involving 810 children and adolescents aged 7 to 17 years who were undergoing mandatory assessments for competitive sports eligibility at the Sports Medicine Unit in Modena. In Italy, engaging in competitive sports necessitates a specialist medical evaluation that includes anthropometric measurements, blood pressure checks, and electrocardiography. Children suffering from chronic systemic diseases that impact growth or metabolism were not included in the study.

### 2.1. Anthropometric Assessment

Height was recorded to the nearest 0.1 cm using a wall-mounted stadiometer, while body weight was measured to the nearest 0.1 kg with a calibrated digital scale, ensuring participants were dressed in light clothing and without shoes. BMI was computed as kg/m^2^ and represented as age- and sex-specific percentiles in accordance with WHO growth reference standards. Waist circumference was taken at the midpoint between the lowest rib and the iliac crest using a non-elastic tape, and the Waist-to-Height Ratio (WHtR) was determined by dividing waist circumference by height. Although no universally accepted WHtR cut-off values exist for children and adolescents, owing to age- and sex-related changes in body proportions during growth and pubertal development, a threshold of 0.5 has been widely used in pediatric research as a pragmatic marker of central adiposity. This approach is supported by several large studies [11,13,14,15]. A 2010 study involving 3091 children aged 4–18 years showed that normal-weight children with central obesity had more adverse cardiometabolic risk profiles than those without central obesity, while overweight or obese children without central obesity exhibited significantly lower risk factor levels; in this study, a WHtR > 0.5 was considered indicative of increased cardiometabolic risk [20]. Similarly, a larger study including 14,493 participants aged 5–18 years demonstrated that increasing WHtR further stratified cardiometabolic risk beyond BMI percentile categories, with BMI-defined weight groups further subdivided according to WHtR (<0.5, 0.5–<0.6, ≥0.6) [21]. On this basis, WHtR > 0.5 was selected in the present study to ensure comparability with the existing literature and to enhance the clinical interpretability of findings.

### 2.2. Blood Pressure Measurement

Resting blood pressure was recorded following a minimum of five minutes of seated rest, utilizing validated pediatric instruments and suitable cuff sizes. Blood pressure readings were categorized based on age-, sex-, and height-specific percentiles, in line with the 2017 guidelines from the American Academy of Pediatrics.

### 2.3. Cardiovascular Knowledge and Lifestyle Assessment

Cardiovascular awareness and lifestyle habits were evaluated through a 20-item questionnaire (Supplementary Materials) that has been previously utilized in pediatric cardiovascular prevention contexts [17,18,19]. The items assessed knowledge regarding physical activity guidelines, dietary risk factors, sedentary behavior, and the link between being overweight and cardiovascular disease. Responses were marked as correct or incorrect, leading to a composite knowledge score calculated as the percentage of correct responses. The internal consistency within the current cohort was deemed acceptable (Cronbach’s α = 0.76). Average daily sedentary time was self-reported and recorded in hours per day [4,9,17].

### 2.4. Statistical Analysis

Data were examined through descriptive statistics, presenting means ± standard deviations (SD) for continuous variables and percentages for categorical variables. Normality was evaluated using Shapiro–Wilk tests. Appropriate statistical tests, such as independent t-tests or Mann–Whitney U tests, were utilized, while chi-square tests assessed associations between categorical variables, with Cramér’s V used to estimate effect sizes (small 0.1, medium 0.3, large 0.5). Pearson correlation coefficients were employed to measure relationships among anthropometric measures, blood pressure, and sedentary behavior.

To address potential confounding factors, analyses extended beyond descriptive or univariable comparisons. Multivariable logistic regression models were developed to investigate the independent associations between anthropometric indicators, lifestyle-related behaviors, and elevated blood pressure. Age and sex were predetermined as significant non-modifiable confounders due to their recognized impact on blood pressure and body composition during growth, with sedentary time included as a behavioral covariate to help mitigate lifestyle-related confounding. Anthropometric variables, such as WHtR and BMI percentile, were simultaneously included in the models to evaluate their relative contributions. Multicollinearity was examined using variance inflation factors, and model calibration was assessed through the Hosmer–Lemeshow goodness-of-fit test. Cardiovascular knowledge scores were analyzed across BMI categories using analysis of covariance (ANCOVA), adjusting for age and sex. Blood pressure analyses were performed using complete-case analysis. Statistical significance was determined at *p* < 0.05.

## 3. Results

A total of 810 children and adolescents participated in the anthropometric analyses. Among them, 389 (48.0%) were female and 417 (51.5%) were male, with sex data missing for four participants (0.5%), who were excluded from the sex-stratified analyses. The average age of the study population was 13.6 ± 2.1 years. The baseline demographic and anthropometric characteristics are detailed in Table 1.

**Figure 1:**
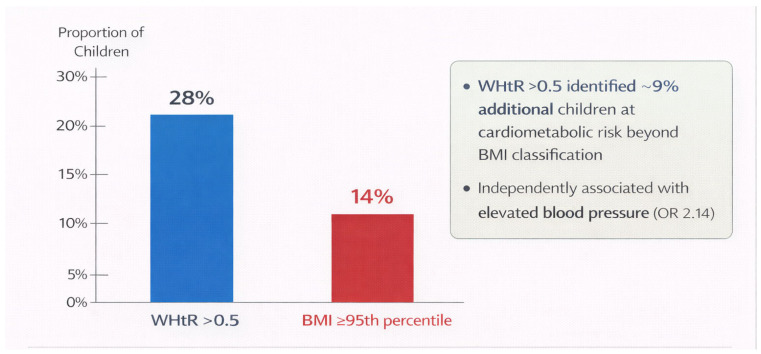
Waist-to-height ratio identifies central adiposity and cardiometabolic risk beyond BMI in children and adolescents.

Mean body mass index was 20.3 ± 3.4 kg/m^2^. According to WHO BMI-for-age percentiles, 64% of participants were classified as normal weight, 22% as overweight, and 14% as obese. A WHtR > 0.5 was observed in 28% of the overall cohort, indicating a high prevalence of central adiposity. Importantly, WHtR identified a subset of children with increased cardiometabolic risk who would not have been classified as overweight or obese based on BMI criteria alone. Approximately 9% of participants with normal BMI percentiles exhibited a WHtR > 0.5, highlighting the added discriminatory value of WHtR beyond BMI classification (Figure 1).

The occurrence of WHtR > 0.5 showed a progressive increase across different BMI categories, being the lowest in normal-weight individuals and the highest in obese children (*p* < 0.001 as per chi-square test), thereby confirming the strong link between excess body mass and central fat accumulation. Resting blood pressure readings were obtained for 769 participants (94.9%). Elevated blood pressure, defined based on age, sex, and height-specific percentiles, was found in 12% of the assessed group. The prevalence of elevated blood pressure was notably higher among those with excess body fat. In particular, children categorized as overweight or obese had a greater percentage of elevated blood pressure compared to their normal-weight counterparts (χ^2^ = 7.21, *p* < 0.01), with a moderate effect size (Cramér’s V = 0.27), suggesting a clinically significant relationship (Table 2).

**Figure 2:**
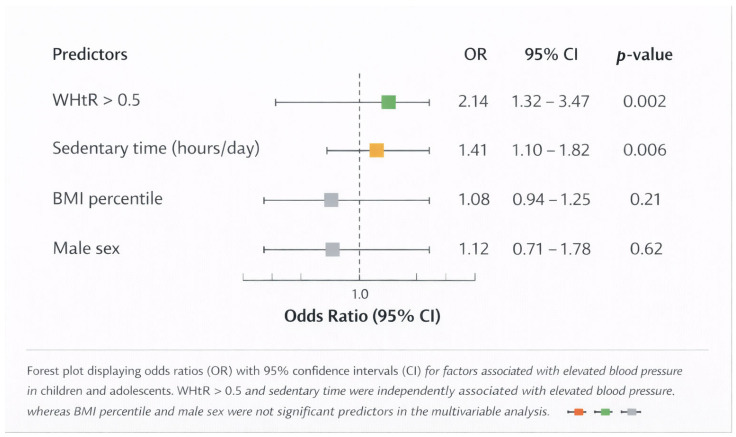
Determinants of elevated blood pressure in pediatric population.

When categorized by central adiposity, individuals with a WHtR greater than 0.5 exhibited a significantly higher occurrence of elevated blood pressure in comparison to those with a WHtR of 0.5 or less (*p* < 0.001), highlighting the importance of central fat distribution in the early rise in blood pressure. To evaluate the independent impact of anthropometric and lifestyle factors on elevated blood pressure, multivariable logistic regression analyses were conducted, controlling for age and sex (Table 2; Figure 2).

In these models, a WHtR greater than 0.5 was identified as an independent predictor of increased blood pressure (OR 2.14, 95% CI 1.32–3.47, *p* = 0.002). Furthermore, sedentary time was found to be independently linked to elevated blood pressure, showing a 41% rise in odds for each additional hour spent sedentary per day (OR 1.41, 95% CI 1.10–1.82, *p* = 0.006).

**Figure 3:**
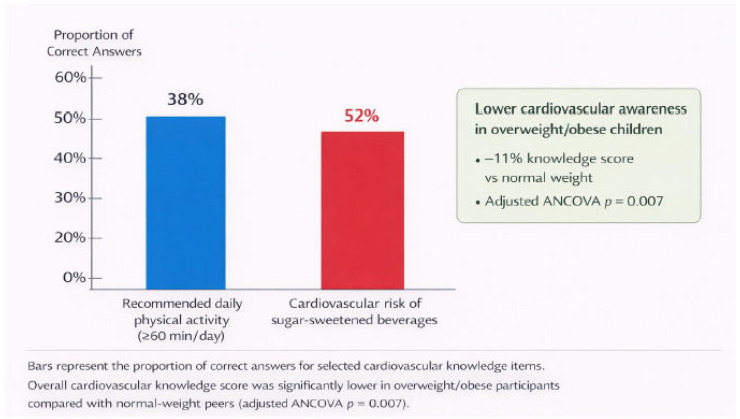
Cardiovascular health awareness in the study cohort.

Conversely, the BMI percentile did not maintain statistical significance when WHtR was factored into the model, indicating that central adiposity, rather than total body mass, is the key anthropometric factor influencing early blood pressure increases in this group. No signs of multicollinearity were observed among the predictors, and the model fit was deemed satisfactory. The baseline knowledge of cardiovascular health was generally low throughout the cohort. Merely 38% of participants accurately recognized the recommended daily physical activity levels, while 52% underestimated the cardiovascular risks linked to high consumption of sugar-sweetened beverages (Figure 3). Cardiovascular knowledge scores varied significantly across different BMI categories. Overweight and obese individuals exhibited notably lower knowledge scores compared to their normal-weight counterparts (mean difference 11%, *p* < 0.05). This relationship remained statistically significant even after adjusting for age and sex through analysis of covariance (F = 7.43, *p* = 0.007), suggesting that lower cardiovascular awareness is associated with excess body fat, independent of demographic variables.

## 4. Discussion

In this study we present robust evidence supporting the combination of anthropometric screening and educational initiatives for early cardiovascular prevention in children and adolescents. In a large group being assessed for competitive sports eligibility, WHtR was found to be a particularly sensitive measure of early cardiometabolic risk, surpassing BMI in its ability to identify children with high blood pressure and central fat accumulation. Concurrently, the study reveals significant deficiencies in cardiovascular health knowledge, particularly among overweight and obese individuals, highlighting the essential role of education in influencing early risk patterns.

Although cardiovascular risk in childhood is influenced by multiple interacting biological and behavioral factors, the present study employed multivariable analytical approaches to reduce confounding and improve interpretability. By simultaneously accounting for age, sex, anthropometric measures, and sedentary behavior, our analyses move beyond purely descriptive observations and allow identification of independent associations relevant to early cardiovascular prevention.

A key finding of this research is the high occurrence of central adiposity, indicated by a WHtR greater than 0.5 in almost one-third of the participants. Notably, WHtR pinpointed a clinically significant subgroup of children who had normal BMI percentiles yet exhibited increased central fat accumulation (Figure 1). This finding corroborates and expands upon previous research indicating that BMI alone may not adequately reflect cardiometabolic risk in pediatric populations, as it fails to account for fat distribution or visceral fat [11,12,13,14,15]. Central adiposity is metabolically active and is crucial in the pathophysiology of early cardiovascular risk through mechanisms that include chronic low-grade inflammation, altered adipokine secretion, insulin resistance, endothelial dysfunction, and heightened arterial stiffness [7,8,9]. These mechanisms can hasten vascular aging and lead to early increases in blood pressure, even when overt obesity is not present.

Children exhibiting a WHtR greater than 0.5 showed a notably higher prevalence of elevated blood pressure, a correlation that remained strong even after adjusting for age and sex in multivariable logistic regression analyses (Table 2; Figure 2). Importantly, when WHtR was factored into the model, the BMI percentile lost its statistical significance, indicating that fat distribution, rather than total body mass, is the key anthropometric factor influencing early blood pressure increases in this demographic. These results are consistent with the accumulating evidence that WHtR serves as a straightforward, age-independent, and highly effective screening method for early cardiometabolic risk assessment in children and adolescents [11,12,13,15].

The conclusions of this study are intentionally framed within a preventive and screening-oriented perspective rather than causal inference. Given the cross-sectional design, our findings do not aim to establish causal pathways but to identify early markers of vulnerability that may be actionable in real-world pediatric settings. Within this framework, the observed independent associations provide clinically meaningful information consistent with the objectives of early cardiovascular prevention.

In addition to anthropometric variables, lifestyle behaviors were found to be significant factors contributing to early cardiovascular risk. Increased sedentary time was independently linked to higher blood pressure, with a gradual rise in risk associated with each additional hour of daily sedentary activity. This observation underscores the notion that physical inactivity and extended periods of sedentary behavior have harmful effects on vascular health, irrespective of adiposity [20,22,23,24]. Sedentary habits encourage visceral fat accumulation, hinder insulin sensitivity, and lead to autonomic imbalance and vascular dysfunction, thereby heightening cardiometabolic risk during crucial developmental stages.

Equally alarming is the insufficient baseline knowledge of cardiovascular health found in this group. Even after participating in sports medical evaluations, less than half of the individuals showed a proper understanding of the suggested levels of physical activity, dietary risks, or the connection between obesity and cardiovascular disease (Figure 3). Notably, cardiovascular knowledge scores were considerably lower in overweight and obese children, even when accounting for age and sex. This combination of biological risk and diminished health literacy indicates a two-way relationship where a lack of awareness can lead to unhealthy behaviors, while excess body fat may further entrench negative lifestyle habits.

The significance of these findings is further emphasized by recent data from Italy, which shows a continuous decrease in physical activity and sports participation among youth following the COVID-19 pandemic, along with a rise in sedentary behavior [25]. Despite the current cohort being a positively selected group—children and adolescents who qualify for competitive sports—the occurrence of central adiposity, high blood pressure, and insufficient cardiovascular knowledge remains considerable. This indicates that the early cardiometabolic risk burden in the general pediatric population is likely even more pronounced.

Sports medicine evaluations offer a distinctive and underused opportunity for early cardiovascular prevention. These assessments facilitate structured, repeated interactions with healthcare professionals and enable the simultaneous assessment of anthropometric risk factors while delivering targeted educational messages. The incorporation of routine Waist-to-Height Ratio (WHtR) measurement into sports eligibility screening is particularly attractive due to its ease of use, affordability, and strong correlation with cardiometabolic outcomes. Combining anthropometric screening with concise, structured educational interventions may increase awareness, encourage healthier lifestyle choices, and support early behavioral changes.

From a wider public health standpoint, the results of this study advocate for a proactive, multidimensional strategy for pediatric cardiovascular prevention. Educational initiatives carried out in schools, sports environments, and family settings have demonstrated effectiveness in enhancing physical activity levels, dietary habits, and cardiometabolic profiles [24,26,27,28]. Even modest early lifestyle changes can lead to significant physiological advantages, such as improved insulin sensitivity, decreased inflammatory burden, and better endothelial function, which have long-term benefits for cardiovascular health.

This study supports existing international guidelines that emphasize the importance of early detection of cardiometabolic risk and the encouragement of healthy behaviors starting in childhood [4,5,6,17,18,19]. The integrated evaluation of central adiposity, blood pressure, lifestyle choices, and cardiovascular knowledge provides a thorough framework for early risk assessment and intervention. By promoting awareness and healthy practices from a young age, these strategies can significantly influence lifelong cardiovascular outcomes and lessen the future impact of cardiovascular disease.

## 5. Limitations

This study has several limitations. Its cross-sectional design precludes causal inference and does not allow assessment of temporal relationships. Residual confounding cannot be fully excluded, as variables such as dietary intake, pubertal stage, socioeconomic status, and family cardiovascular history were not systematically collected. Lifestyle behaviors were self-reported and may be subject to reporting bias, and the population was limited to children undergoing sports eligibility screening, which may affect generalizability. Waist-based anthropometric indices can also vary across pubertal stages and between sexes, potentially influencing WHtR-based risk classification.

Nonetheless, the large sample and comprehensive assessment of anthropometric and behavioral variables, together with multivariable modeling adjusting for major non-modifiable and behavioral confounders, strengthen the robustness of the observed associations and support early identification of at-risk children. Although the questionnaire was not a formal psychometric instrument, its acceptable internal consistency supports its use for estimating baseline cardiovascular knowledge. Longitudinal studies with objective activity monitoring, metabolic profiling, and cardiovascular imaging are warranted to assess the long-term impact of early interventions.

## 6. Future Directions

Future research and clinical practice should encourage the regular implementation of waist-based anthropometric measurements in pediatric healthcare to enhance early cardiometabolic risk evaluation. Evidence indicates that the waist-to-hip ratio (WHR) is a more effective indicator of central adiposity compared to BMI alone and is linked to components of metabolic syndrome, such as insulin resistance and altered glucose metabolism, thereby underscoring its importance for cardiovascular and diabetes risk assessment in children and adolescents [29]. The introduction of new technologies, including automated and non-contact assessment methods, could significantly improve the feasibility and accuracy of WHR measurement in youth populations [30]. Longitudinal research is essential to determine the developmental pathways of clinically significant thresholds for waist-based anthropometric measures throughout growth and puberty.

## 7. Conclusions

The early identification of cardiometabolic risk in children can be effectively achieved through straightforward anthropometric measures, especially the WHtR. There is a notable lack of baseline cardiovascular knowledge, particularly in overweight and obese children, which highlights the critical need for educational initiatives. It is vital to implement educational programs that merge anthropometric assessments with the encouragement of physical activity, healthy eating, and cardiovascular health awareness. Such initiatives are crucial for promoting healthy habits, ensuring immediate well-being, and preventing future cardiovascular diseases [24,27,28]. These findings support the potential role of waist-to-height ratio as a practical and sensitive screening tool for early cardiometabolic risk identification in children and adolescents. Integrating anthropometric screening with lifestyle assessment and education may represent a feasible strategy to enhance early cardiovascular prevention.

## Figures and Tables

**Table 1:** Baseline demographic and anthropometric characteristics of the study cohort.

Variable	Value
Participants, n	810
Age, years (mean ± SD)	13.6 ± 2.1
Sex, n (%)	Female 389 (48.0); Male 417 (51.5); Missing 4 (0.5) *
BMI, kg/m^2^ (mean ± SD)	20.3 ± 3.4
Weight status (WHO BMI-for-age), %	Normal weight 64; Overweight 22; Obesity 14
WHtR > 0.5, %	28
Blood pressure available, n	769
Elevated blood pressure †, %	12

* Four participants had missing sex data in sex-stratified analyses, as reported. † Classified according to 2017 American Academy of Pediatrics age-, sex-, and height-specific percentiles.

**Table 2:** Key associations reported in univariable analyses.

Association (Exposure → Outcome)	Direction (as Reported)	Test/Metric Reported	*p*-Value
Overweight/obesity → Elevated BP	Higher prevalence with excess adiposity	χ^2^ = 7.21; Cramér’s V = 0.27	<0.01
Overweight/obesity → WHtR > 0.5	More frequent in overweight/obese	Chi-square	<0.001
Overweight/obesity → Cardiovascular knowledge score	Lower score vs normal weight	Group comparison (adjusted analysis reported separately)	<0.05

## Data Availability

The data presented in this study are available on request from the corresponding author.

## Supplementary Materials

The following supporting information can be downloaded at: https://www.mdpi.com/article/10.3390/jcdd13010057/s1, Questionnaire Items.
