# Supplementary material for: Anthropometric Indicators and Early Cardiovascular Prevention in Children and Adolescents: The Role of Education and Lifestyle
Source: J Cardiovasc Dev Dis. 2026 Jan 22;13(1):57. doi: 10.3390/jcdd13010057 (PMC12841735; doi:10.3390/jcdd13010057)
Supplement: Supplementary file 1 [file jcdd-13-00057-s001.zip › jcdd-4082386-supplementary.pdf]

Supplementary Material S1

Cardiovascular Health Awareness Questionnaire for Children and Adolescents

Purpose

This questionnaire assesses baseline knowledge of cardiovascular health, lifestyle-related risk factors, and prevention strategies in children and adolescents aged 7–17 years. It was used for descriptive and educational purposes and is not intended as a formal psychometric instrument.

Questionnaire Items

Section 1 – Physical activity

1. How much physical activity should children and adolescents perform every day to stay healthy?

a) Less than 30 minutes

b) About 30 minutes

c) At least 60 minutes

d) Only on sports days

2. Regular physical activity helps keep the heart healthy. (True / False)

3. Which of the following is an example of physical activity?

a) Watching television

b) Cycling or running

c) Using a smartphone

d) Playing video games

Section 2 – Sedentary behaviour

4. Spending many hours sitting in front of screens can be harmful to health. (True / False)

5. Prolonged sedentary behaviour may increase the risk of heart problems over time. (True / False)

6. Which is a good way to reduce sedentary time?

a) Sleeping more

b) Taking active breaks and moving during the day

c) Using electronic devices more

d) Watching more television

Section 3 – Nutrition

7. Frequently drinking sugar-sweetened beverages is good for heart health. (True / False)

8. Sugar-sweetened beverages can increase the risk of overweight and cardiovascular problems. (True / False)

9. Which food choice is healthier for the heart?

a) Packaged snacks

b) Sugar-sweetened beverages

c) Fruit and vegetables

d) Sweets

Section 4 – Body weight and obesity

10. Being overweight or obese can increase the risk of heart problems even at a young age. (True / False)

11. Fat accumulation mainly in the abdominal area is more harmful to heart health. (True / False)

12. Which measure helps identify abdominal fat accumulation?

a) Body weight

b) Waist-to-height ratio

c) Age

d) Height

Section 5 – Blood pressure

13. Blood pressure is the force of blood flowing through blood vessels. (True / False)

14. Having elevated blood pressure in childhood may affect heart health later in life. (True / False)

15. Blood pressure levels can be influenced by lifestyle habits. (True / False)

Section 6 – General cardiovascular risk

16. Cardiovascular diseases affect only elderly people. (True / False)

17. A healthy lifestyle can reduce the risk of cardiovascular diseases. (True / False)

Section 7 – Prevention and education

18. Developing healthy habits during childhood is important for future heart health. (True / False)

19. Some cardiovascular risk factors can be improved by changing lifestyle habits. (True / False)

20. Learning about heart health at school and during sports activities is useful for disease prevention. (True / False)

Scoring

Each correct answer scores 1 point. The overall knowledge score is expressed as the percentage of correct responses. The questionnaire showed acceptable internal consistency in the study cohort (Cronbach's α = 0.76).
